# Molecular Networking for Drug Toxicities Studies: The Case of Hydroxychloroquine in COVID-19 Patients

**DOI:** 10.3390/ijms23010082

**Published:** 2021-12-22

**Authors:** Pierre-Jean Ferron, Brendan Le Daré, Julie Bronsard, Clara Steichen, Elodie Babina, Romain Pelletier, Thierry Hauet, Isabelle Morel, Karin Tarte, Florian Reizine, Bruno Clément, Bernard Fromenty, Thomas Gicquel

**Affiliations:** 1 INSERM, Univ Rennes, INRAE, Institut NuMeCan (Nutrition Metabolisms and Cancer) UMR_A 1341, UMR_S 1241, Previtox Network, 35000 Rennes, France; pierre-jean.ferron@inserm.fr (P.-J.F.); brendan.le.dare@chu-rennes.fr (B.L.D.); julie.bronsard@inserm.fr (J.B.); elodiebabina@gmail.com (E.B.); isabelle.morel@chu-rennes.fr (I.M.); bruno.clement@inserm.fr (B.C.); bernard.fromenty@inserm.fr (B.F.); 2 Pôle Pharmacie, Service Hospitalo-Universitaire de Pharmacie, CHU Rennes, 35000 Rennes, France; 3 INSERM U1082 (IRTOMIT), 86000 Poitiers, France; clara.steichen@univ-poitiers.fr (C.S.); thierry.hauet@gmail.com (T.H.); 4 Faculté de Médecine et Pharmacie, Université de Poitiers, 86000 Poitiers, France; 5 Laboratoire de Toxicologie Médico-Légale, CHU Rennes, 35000 Rennes, France; romain.pelletier@chu-rennes.fr; 6 Service de Biochimie, CHU Poitiers, 86000 Poitiers, France; 7 UMR 1236, Univ Rennes, INSERM, Etablissement Français du Sang Bretagne, 35043 Rennes, France; karin.tarte@univ-rennes1.fr (K.T.); florian.reizine@chu-rennes.fr (F.R.); 8 Laboratoire SITI, CHU Rennes, 35033 Rennes, France; 9 Maladies Infectieuses et Réanimation Médicale, CHU Rennes, 35033 Rennes, France

**Keywords:** molecular networking, hydroxychloroquine, drug metabolism, HepaRG, COVID-19, fatty liver

## Abstract

Using drugs to treat COVID-19 symptoms may induce adverse effects and modify patient outcomes. These adverse events may be further aggravated in obese patients, who often present different illnesses such as metabolic-associated fatty liver disease. In Rennes University Hospital, several drug such as hydroxychloroquine (HCQ) have been used in the clinical trial HARMONICOV to treat COVID-19 patients, including obese patients. The aim of this study is to determine whether HCQ metabolism and hepatotoxicity are worsened in obese patients using an in vivo/in vitro approach. Liquid chromatography high resolution mass spectrometry in combination with untargeted screening and molecular networking were employed to study drug metabolism in vivo (patient’s plasma) and in vitro (HepaRG cells and RPTEC cells). In addition, HepaRG cells model were used to reproduce pathophysiological features of obese patient metabolism, i.e., in the condition of hepatic steatosis. The metabolic signature of HCQ was modified in HepaRG cells cultured under a steatosis condition and a new metabolite was detected (carboxychloroquine). The RPTEC model was found to produce only one metabolite. A higher cytotoxicity of HCQ was observed in HepaRG cells exposed to exogenous fatty acids, while neutral lipid accumulation (steatosis) was further enhanced in these cells. These in vitro data were compared with the biological parameters of 17 COVID-19 patients treated with HCQ included in the HARMONICOV cohort. Overall, our data suggest that steatosis may be a risk factor for altered drug metabolism and possibly toxicity of HCQ.

## 1. Introduction

Coronavirus disease 2019 (COVID-19) outbreak is caused by infection with severe acute respiratory syndrome coronavirus 2 (SARS-CoV-2 virus). Symptoms of COVID-19 include acute respiratory infection (fever, cough, and shortness of breath), which can lead to acute respiratory distress syndrome (ARDS) and requires specific management in the intensive care unit (ICU) [1]. The pathophysiology of SARS-CoV-2 infection is close to SARS-CoV-1 infection, characterized by damage to the airways with aggressive inflammatory responses [2]. Although most patients are asymptomatic, some patients also show signs of hepatotoxicity, including an increase of transaminases (alanine aminotransferase (ALT) and aspartate aminotransferase (AST)) and γ-glutamyltransferase (GGT) [3,4]. Moreover, studies suggest that the presence of the ACE2 receptor on cholangiocytes could label them as potential targets for SARS-CoV-2 infection and induce disturbances in the bile acid metabolism [5]. In addition to liver damage, some articles have also reported an increased incidence of acute kidney injury as a result of COVID-19, which could be due to a systemic inflammatory reaction and also to the direct presence of SARS-CoV-2 in kidney tissue [6]. An increase in mortality has been observed in patients with kidney dysfunction, but the exact cause cannot be clarified [7,8].

The liver and kidney are highly sensitive to hypoxia and, moreover, the significant inflammation linked to the acute respiratory infection could induce a major production of pro-inflammatory cytokines deleterious for their functions (an event also referred to as the “cytokine storm”) [9]. In addition, a higher frequency of hepatic steatosis has been found in COVID-19 positive individuals [10,11], and liver injury and obesity are frequently reported in patients with severe COVID-19 symptoms [12]. These comorbidities are often correlated with metabolic-associated fatty liver disease (MAFLD), and may be expose to impaired drug metabolism and subsequent drug-induced liver injury [13]. Thus, hepatic and renal alterations can be induced by both COVID-19 and drug-induced toxicity. Several treatments are currently being considered to fight SARS-CoV-2. These treatments are known (or suspected) to induce liver damage in some COVID-19 patients [3]. Among these drugs, hydroxychloroquine (HCQ) has been evaluated in the English clinical trial “Recovery” [14] and the French clinical trial “Discovery” [15].

HCQ is a 4-aminoquinoline marketed as Plaquenil^®^, and is used in the treatment for malaria and rheumatic and inflammatory diseases (rheumatoid arthritis and lupus erythematosus). HCQ has anti-inflammatory and analgesic actions, which may be of benefit during SARS-CoV-2 infection. Interestingly, recent studies hypothesizes that HCQ modifies endosomes pH and inactivates viruses during their infections [16,17]. The cytochromes P450 2D6 and 3A4 (CYP2D6 and CYP3A4) participate in the N-dealkylation of HCQ to the active metabolite, desethylhydroxychloroquine (DHCQ), as well as in the generation of the inactive metabolites, desethylchloroquine (DCQ), and bidesethylchloroquine [18,19]. DHCQ is the main metabolite found in the blood and urine [20,21]. Chloroquine has demonstrated antiviral effects, in particular on the viral replication of several coronaviruses, including SARS-CoV and MERS-CoV [21,22]. HCQ inhibits infection of Vero6 cells by SARS-CoV-2 in vitro with an absence of toxicity up to 100 µM [17]. However, recent studies showed inconsistent results regarding HCQ efficacy to treat COVID-19 [14,23]. In this context, the question of impaired HCQ metabolism and pharmacokinetics under certain pathological conditions arises, and could provide valuable information explaining the differences in efficacy observed in vitro and in patients [24]. Thus, HCQ metabolism studies in COVID-related pathological conditions would be of particular interest.

Recently, molecular networking has emerged as a powerful tool in order to explore metabolism, either in vivo or in vitro. This bioinformatics tool allows for the organization and representation of untargeted tandem mass spectrometry (MS/MS) data in a graphical form [25]. Each detected ion can be linked to other compounds according to their spectral similarities, thus facilitating metabolite identification [26]. Moreover, by providing valuable insights into sample-to-sample comparison, the molecular networking approach offers relevant information concerning metabolic profiles [26,27]. As in vitro cellular models are relevant to study drug metabolism in normal and pathological conditions, molecular networking might be a promising tool to compare the metabolic profiles of COVID-19 treatment between these conditions.

In this study, we aimed to assess HCQ molecular networking and the toxicological profile by using the human kidney cell line RPTEC and the human hepatocyte cell line HepaRG in an original in vivo/in vitro approach. Noteworthy, these cellular models are commonly used to study drug-induced nephrotoxicity and hepatotoxicity in vitro, respectively [28,29,30]. Moreover, we wished to determine whether fatty acid treatment could impair HCQ metabolism and cytotoxicity in the HepaRG cells. Indeed, we previously successfully used this approach in order to demonstrate that the cytotoxicity of specific xenobiotics could be increased in the context of fatty acid overload [31,32,33]. Finally, we also aimed to evaluate HCQ metabolism in COVID-19 patients in comparison with their biological characteristics.

## 2. Results

### 2.1. Cytotoxicity and Metabolism of Hydroxychloroquine

**Figure 1:**
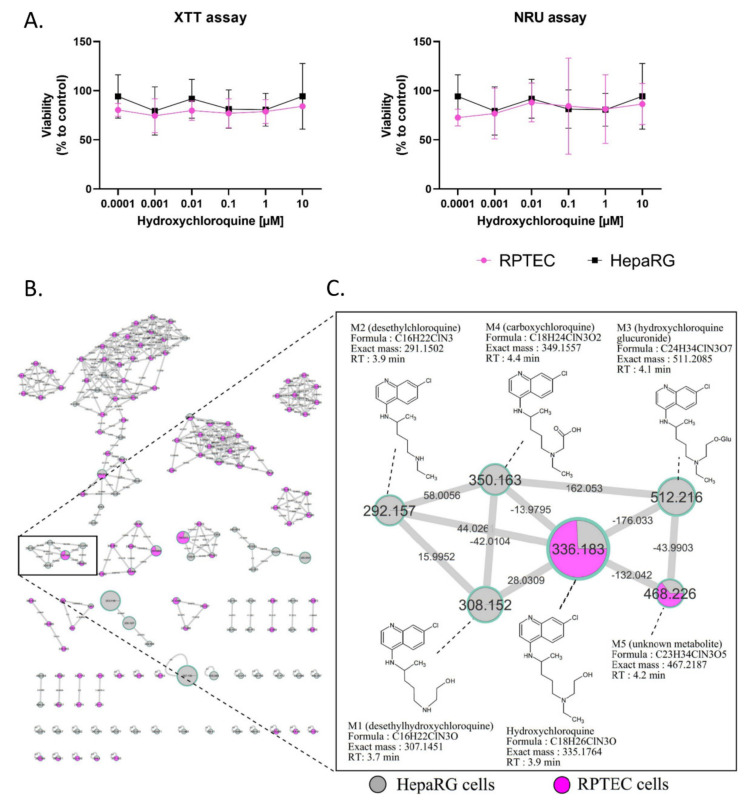
Cytotoxicity and visualization of in vitro HCQ metabolism using molecular networking. Differentiated HepaRG and RPTEC were incubated with HCQ (10 µM) for 48h (at least three experiments in both cell lines). (**A**) Cytotoxicity was evaluated using XTT and NRU assays. Cell viability was calculated compared to control conditions after 48 h of treatment. (**B**) The molecular network. Each cell type is depicted in a specific color: HepaRG cells in grey and RPTEC in pink. (**C**) Details of the specific HCQ-containing cluster. Nodes are labelled with the exact protonated mass (*m/z*) and the links are labelled with the exact mass shift. Proposed metabolites of HCQ structure are linked to the corresponding nodes.

RPTEC and HepaRG cells were treated for 48 h with HCQ (0.0001 to 10 µM). The highest HCQ concentration tested was two-fold above the maximal EC50 observed during clinical evaluation of the HCQ antiviral activity. The cytotoxicity of HCQ was evaluated with both XTT and NRU assays, and showed no cytotoxic effects on both cell lines (Figure 1A).

No changes in RPTEC and HepaRG morphology were noticed in bright field microscopy (data not shown) compared to the control condition. Based on the absence of toxic effects at the highest concentration tested, HCQ metabolite screening was performed with 10 µM HCQ. Culture media analysis using untargeted liquid chromatography high resolution mass spectrometry (LC-HRMS/MS) screening allowed us to generate a multi-matrix molecular network (Figure 1B,C). All MS/MS data acquired during the analysis are displayed, and a specific color was assigned to each cell culture type (HepaRG cells in grey and RPTEC in pink). Nodes were linked together according to their MS/MS spectral similarities (Figure 1B). Multi-matrix molecular network visual analysis found a cluster containing HCQ (*m/z* 336.183, RT: 3.9 min), linked to five other nodes (M1: *m/z* 308.152 (RT: 3.7 min), M2: *m/z* 292.157 (RT: 3.9 min), M3: *m/z* 512.216 (RT: 4.1 min), M4: *m/z* 350.163 (RT: 4.4 min), and M5: *m/z* 468.226 (RT: 4.2 min)) (Figure 1C). Four of these nodes are linked to HCQ, with well-established biotransformation mass shifts of 28.031, 44.0261, 176.033, and 13.9795, corresponding to deethylation, deacetylation + dehydrogenation, glucuronidation, and C terminal oxidation + dehydrogenation, respectively (Supplementary Table S1). The identification step revealed that these compounds could correspond for M1 (*m/z* 308.152) to DHCQ, for M2 (*m/z* 512.216) to DCQ, for M3 (*m/z* 511.208) to glucuronide, and for M4 (*m/z* 349.155), a new metabolite identify as carboxychloroquine (Figure 1C). Interestingly, these four putative metabolites were only found in theHepaRG cell supernatant. In addition, the M5 (*m/z* 468.226) node was linked to HCQ with a mass shift of + 132.042, corresponding to the addition of a C5H8O4 group. This compound was found in both RPTEC and HepaRG cells. The LC-HRMS/MS parameters of these different compounds are presented in Supplementary Table S2. Taken together, these results suggest that (i) HCQ is not cytotoxic in non-pathological in vitro renal and liver tissue models, and (ii) that RPTEC is a low metabolizer of HCQ, conversely to differentiated HepaRG cells. As a result, only differentiated HepaRG cells were kept to further explore HCQ metabolism in pathological conditions.

### 2.2. In Vivo Metabolism of Hydroxychloroquine

**Figure 2:**
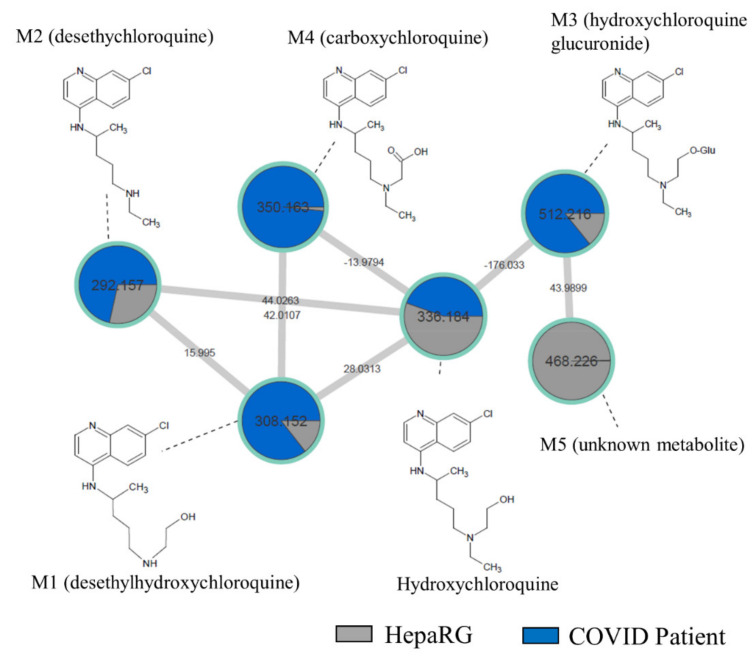
Molecular network comparing metabolites from a non-steatotic COVID-19-positive patient treated with HCQ and “healthy” HepaRG cells. HepaRG cells (grey) were incubated with HCQ (10 µM) for 48 h. The non-steatotic COVID-19-positive patient (dark blue) was treated with HCQ for 4 days at 400 mg/day. Nodes are labelled with the exact protonated mass (*m/z*) and the links are labelled with the exact mass shift. Proposed metabolites of HCQ structure are linked to the corresponding nodes.

In order to compare our in vitro findings to the in vivo data, we performed a sample-to-sample comparison using molecular networking, including a blood sample of COVID-19 patient treated by HCQ and our culture media of differentiated HepaRG incubated by HCQ (10 µM) for 48 h (Figure 2). The HCQ-containing cluster visual analysis revealed that all putative metabolites (M1 to M5) found in our in vitro experiment were present in the patient’s blood sample, highlighting the relevance of the HepaRG cell model in HCQ metabolism study. Among all of the metabolites, the M5 plasma level was, however, very low in this patient.

### 2.3. Fatty Acid Treatment Induces a Change in HCQ Metabolism and Toxicity

**Figure 3:**
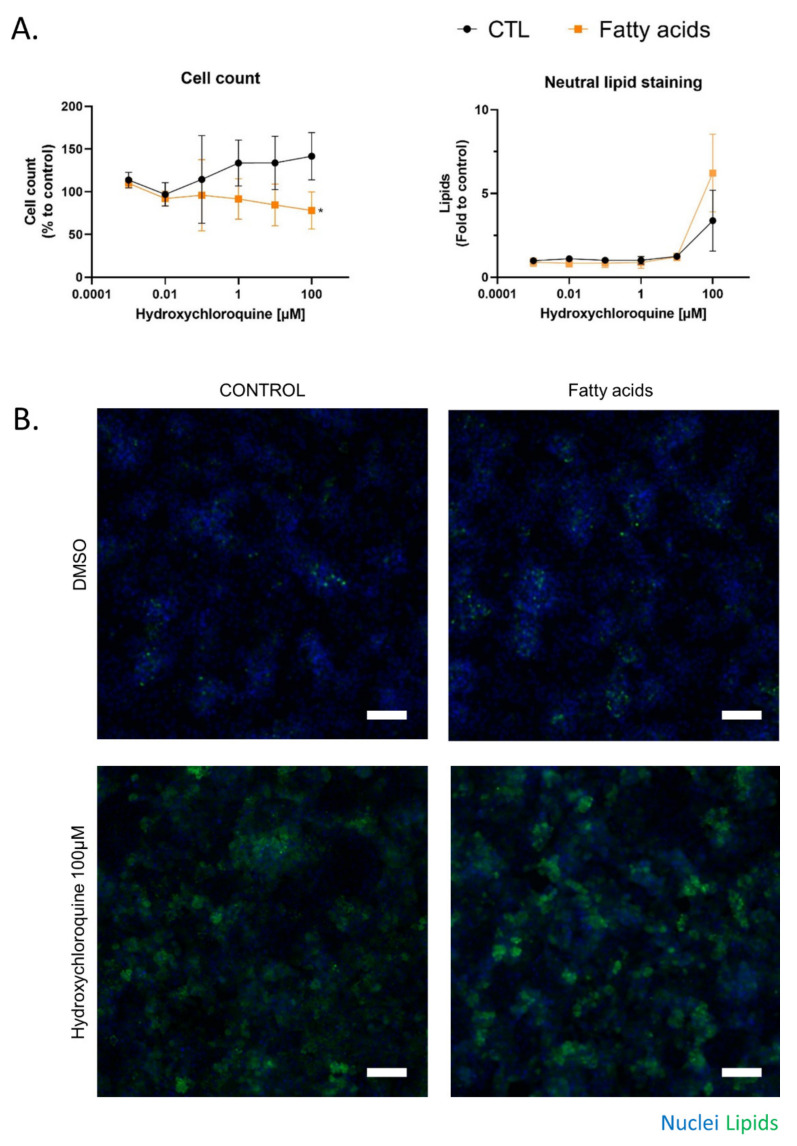
Chronic cytotoxicity of HCQ in HepaRG cells exposed to fatty acids. The effect of HCQ on HepaRG cells under fatty acid overload was evaluated after 10 days of treatment. (**A**) The number of DAPI-stained nuclei in 10 fields per well were counted and compared with vehicle (DMSO 1.7%, control condition). Lipids stained with neutral red were quantified in the cytoplasm of each counted cell and were compared to the control condition. Data represent the mean ± SD of fold changes obtained in three independent experiments performed in triplicate. *T*-test * = *p* < 0.05. (**B**) Representative images at 10× magnification of HepaRG cells treated 10 days with HCQ and fatty acids. DAPI staining in blue corresponds to the nucleus, and Nile red in green correspond to cellular lipids. White scale bar = 100 µm.

HepaRG cells were treated for 10 days with HCQ (0.001 to 100 µM) with or without a mixture of 150 µM of stearic acid and 150 µM of oleic acids. This fatty acids mixture induced a metabolic associated fatty liver disease syndrome in HepaRG cells, characterized by modulation of major CYP activities and the accumulation of intra cellular triglycerides, as previously described [32,33]. This mixture was chosen in our study to allow for the observation of additional lipid accumulation in the cells. Indeed, although we can increase the initial steatosis by increasing the amount of oleic acid, this makes it difficult to observe additional lipid accumulation (Supplementary Figure S1). By using the cell count to assess cytotoxicity, 100 µM HCQ was found to be cytotoxic in fatty acid-treated HepaRG cells, whereas HCQ was not cytotoxic in the control condition (Figure 3A). We next used Nile red, which is known to stain neutral lipids (triglycerides and cholesterol esters) but not phospholipids at emission/fluorescence wavelengths 531/593 nm [34].

**Figure 4:**
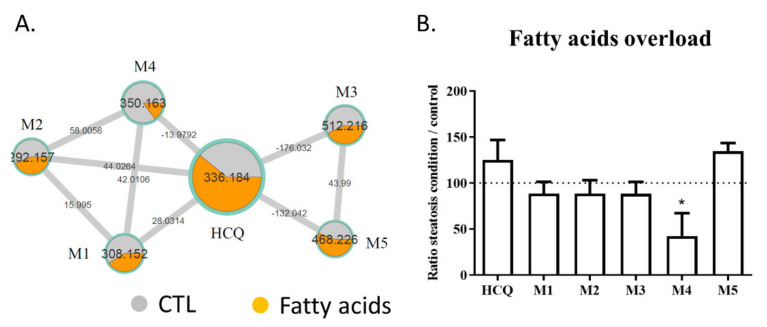
Levels of HCQ and its metabolites detected in the culture medium of HepaRG cells treated or not with fatty acids. The metabolism of HCQ incubated on HepaRG cells without (control condition) or with fatty acids overload was evaluated after 10 days of treatment. (**A**) Details of the specific hydroxychloroquine-containing cluster. Nodes are labelled with the exact protonated mass (*m/z*) and the links are labelled with the exact mass shift. (**B**) Ratio of the peak area of each compound (HCQ, M1 to M5) in the condition of fatty acid treatment to the peak area of each compound in control condition. M1: desethylhydroxychloroquine; M2: desethylchloroquine; M3: hydroxychloroquine glucuronide; M4: carboxychloroquine; M5: Unknown metabolite. The data are quoted as the mean ± SEM from three independent experiments performed in triplicate. Intergroup differences were tested in a two-way ANOVA. * *p* < 0.05 for fatty acids overload condition compared with the control condition for each compound (arbitrary set to 100%).

Lipid staining with Nile red revealed that 100 µM HCQ induced steatosis, whereas a greater neutral lipid accumulation was observed when the HepaRG cells were cultured in the presence of fatty acids (Figure 3A). An observation of Nile red labeling in Figure 3 and in Supplementary Figure S2 shows us that the addition of fatty acid on HepaRG cells induces a “basal mild steatosis”, which worsens with the addition of hydroxychloroquine. However, quantitative analyses of Nile red immunofluorescence did not reveal statistical differences. No changes in HepaRG cell morphology were noticed with fluorescence microscopy compared to the control condition (Figure 3B). To investigate whether fatty acid overload can alter metabolism, HCQ was incubated in differentiated HepaRG cells with or without fatty acids during 10 days. Culture media analysis using untargeted LC-HRMS/MS screening allowed us to generate a molecular network where a specific color was assigned to each condition (control in grey and fatty acid treatment in orange; Figure 4A). Using semi-quantitative analysis, we identified the same five metabolites, but found that the fatty acid treatment was able to modify the HCQ metabolism profile, especially for carboxychloroquine (M4), whose level was apparently decreased (Figure 4A). A significantly lower concentration of carboxychloroquine in the condition of fatty acid overload was confirmed using metabolite peak area comparison in three independent experiments performed in triplicate (Figure 4B). Taken together, these results suggest that the HCQ metabolism signature is modified in the condition of fatty acid overload and is associated with cytotoxicity.

### 2.4. Comparison of HCQ Metabolization between In Vitro and Patients

Based on our in vitro observations, we investigated whether patients’ characteristics (summarized in Table 1) were associated with changes in the metabolite ratios. Analyses were performed on a cohort of 17 COVID-19 patients treated with HCQ after 4 days post-admission. This cohort included 12 patients with ARDS and 5 patients without ARDS. The biological characteristics of the patients are provided in Table 1.

Blood samples were obtained at D4 and plasma samples were analyzed using untargeted LC-HRMS/MS followed by molecular network analysis. The relative level of each metabolite was compared to the patient’s biological characteristics using Pearson correlation (Table 2).

**Figure 5:**
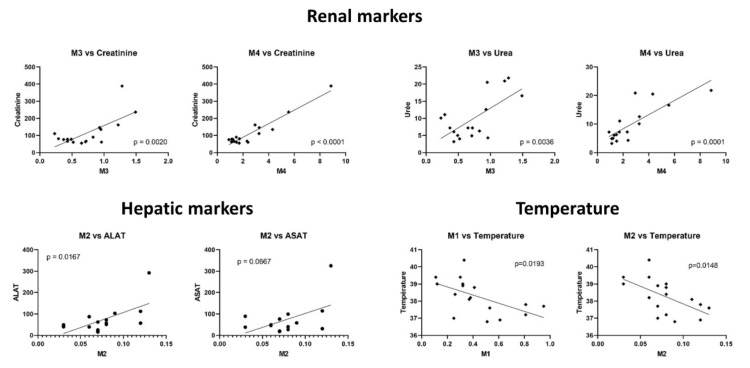
Linear regression between metabolite ratios and biological characteristics. The ratio of the peak area of each compound (HCQ, M1 to M5) in the patient plasma to the peak area of each compound in the control condition is represented as scatter plot. Linear correlation and *p*-value were calculated using GraphPad Prism.

Considering the markers of renal function, the urea and creatinine were positively correlated with a higher level of M3 and M4 in patient samples (Pearson correlation coefficient between 0.67 and 0.95), thus suggesting a renal elimination. Positive linear correlations were found between M2 level and both AST and ALT activities (Pearson correlation coefficient of 0.5 and 0.61, respectively). Temperature was negatively correlated with lower levels of M1 and M2 in patient samples (Pearson correlation coefficient between -0.56 and 0.59, respectively). Linear correlations are plotted in Figure 5, which also shows the respective *p* values. Linear correlations between the M5 level and PaO2 or procalcitonin (PCT), which were possibly biased because of one extreme value of M5 in the patient cohort, are shown in Supplementary Figure S3.

## 3. Discussion

Pathophysiological conditions are increasingly recognized as factors altering drug metabolism. In this study, we showed, using molecular networking, that the condition of fatty acid overload is able to impair hepatocellular HCQ metabolism and toxicity in vitro. These results call for caution in the management of patients with severe COVID-19 symptoms who often suffer from obesity and related metabolic disorders such as MAFLD, although further studies are needed [12,13].

HCQ appeared to be a suitable candidate drug to test this proof of concept. Indeed, this molecule undergoes an extensive metabolism, which is well described in the literature, thus allowing us to assess the relevance of our data [18,35,36]. In addition, HCQ has been evaluated in large clinical trials for the treatment of COVID-19, in particular in our hospital (CHU Rennes, n°35RC20_9795_HARMONICOV, ClinicalTrials.gov Identifier: NCT04373200). Samples from patients treated with HCQ were thus easily available to compare the clinical data with our in vitro study.

Molecular networking was used for metabolite identification, as we already reported its relevance both in vivo and vitro [26,27,37,38]. First, we were interested in comparing the liver and kidney cellular models for the study of HCQ metabolism. Consistent with previous data reporting the CYP-dependent metabolism of HCQ in liver [39], we were able to detect in the human hepatic cell line HepaRG a higher number of metabolites compared to the human kidney cell line RPTEC. Indeed, we identified five putative HCQ metabolites using HepaRG cells, but only one metabolite (M5) using RPTEC. Interestingly, M4 (carboxychloroquine) and M5 (an unknown metabolite) have not already been described. Secondly, we aimed to investigate whether the metabolites found in vitro were also present in vivo. All five metabolites were found in the blood of a COVID-19 patient treated with HCQ, confirming the relevance of the HepaRG cell model for the study of its metabolism.

HepaRG cells have already proven to be useful to study drug-induced hepatic steatosis [28,40]. In this study, 100 µM HCQ induced neutral lipid accumulation in HepaRG cells incubated without fatty acid accumulation. However, to the best of our knowledge, HCQ-induced accumulation of hepatic triglycerides (i.e., steatosis) does not seem to occur in treated patients, contrary to phospholipid accumulation (i.e., phospholipidosis), which is common with this cationic amphiphilic drug [16,41]. Hence, our data suggest that only a few susceptible patients might be at risk for HCQ-induced steatosis. Further investigations will be required to determine whether HCQ impair mitochondrial fatty acid oxidation, de novo lipogenesis, or very low-density lipoprotein secretion, three important mechanisms whereby drugs can induce hepatic steatosis [28,42].

Because obesity and MAFLD might be risk factors for drug-induced liver injury in COVID-19 patients [13], we used HepaRG cells treated with fatty acids to investigate HCQ cytotoxicity and metabolism in the context of basal mild steatosis induced by fatty acids overload, as previously reported for other potential toxic compounds [31,33,40]. Herein, HCQ at the highest tested concentration tended to further aggravate steatosis on the one hand, and was cytotoxic under the condition of fatty acid treatment on the other. We hypothesized that the altered metabolism of HCQ under fatty acid overload might be responsible for its toxicity. Interestingly, the metabolic signature of HCQ was significantly modified in conditions of fatty acid treatment, showing a trend toward an increased HCQ level associated with significantly decreased M4 formation. This suggests that HCQ toxicity in the condition of hepatic lipid overload might be attributed to its accumulation in HepaRG cells. Likewise, aggravation of HCQ-induced steatosis might be secondary to its cellular accumulation. In addition, Boya et al. (2003) reported that HCQ induces mitochondrial membrane permeabilization, as indicated by the insertion of Bax into mitochondrial membranes, the conformational activation of Bax within the mitochondria, the release of cytochrome c from the mitochondria, and the loss of the mitochondrial transmembrane potential [43]. However, a decline in mitochondrial function has been found to provoke metabolic disturbances in animal models and may potentially contribute to MAFLD progression [44]. Therefore, this HCQ-induced mitochondrial damage may also partly explain the worsening of cytotoxicity and hepatic steatosis observed in the presence of fatty acid overload in this study. The precise mechanism(s) whereby HCQ could accumulate and be more cytotoxic in the condition of fatty acid overload would require further investigations. Alternatively, the unknown metabolite M5 might also be cytotoxic, as its levels tended to be enhanced in the condition of fatty acid treatment. Of note, MAFLD is associated with an altered expression and activity of different xenobiotic-metabolizing enzymes such as CYPs and UDP-glucuronosyltransferases (UGTs) [13,32,42]. Beside overweight (or obesity) and MAFLD, other pathophysiological conditions might significantly alter drug metabolism in COVID-19 patients, such as inflammation [45] and fever, as discussed later on.

In this study, 15 patients were overweight or obese, with a BMI above 25 or 30 kg/m^2^, respectively. This might suggest that most of them presented MAFLD, although we did not have liver biopsies in order to confirm this assumption. In COVID-19 patients, we found a statistically significant positive linear correlation between the DCQ (M2) ratio and ALT activity, whereas the *p* value for the AST activity was almost significant (*p* = 0.0667). Further investigations would be needed in order to determine whether, in addition to HCQ and M5, DCQ (M2) could be hepatotoxic by itself, in particular by inducing hepatic cytolysis.

To further study the changes of HCQ metabolism under COVID-19 related pathological conditions, we compared the metabolite ratio of the five HCQ metabolites with different biological characteristics of our patients. Interestingly, M3 (HCQ glucuronide) and M4 (carboxychloroquine) levels were positively correlated with urea and creatinine, suggesting renal elimination. Although the pharmacokinetic feature of glucuronide derivative is well known to be related to renal excretion, for the glucuronide derivative, our data provided novel evidence of renal excretion for the M4 metabolite. Moreover, our data suggested that M3 and M4 renal elimination might be altered in COVID-19 patients with kidney impairment. Alternatively, these metabolites might be toxic for the kidney, or might participate to renal impairment in COVID-19 patients, along with other factors. Finally, body temperature in COVID-19 patients was negatively correlated with decreased M1 (DHCQ) and M2 (DCQ) levels. These results might be consistent with those from Kihara et al. (1998), reporting a decrease in CYP activity during fever, most likely due to the action of secreted pro-inflammatory cytokines [46]. Furthermore, previous studies in our laboratory have shown that hypoxia induces a switch of liver metabolism from aerobic to anaerobic glycolysis and a repression of critical genes as CYP3A4 [47]. These data from the literature could also explain the correlation found between PCT, a marker of an infectious context, and PaO2 with the accumulation of the M5 metabolite (Supplementary Figure S3) [46]. Lastly, cortisol levels were negatively correlated with HCQ. Here, we believe that this context of inflammation does not allow for the metabolic changes observed in vitro by steatosis alone to be observed in patients.

This study presents several limitations. Firstly, we disclosed a modification of HCQ metabolism and toxicity in a cellular model of fatty acid overload [32,33]. However, it would be interesting to perform additional investigations in a cellular model of nonalcoholic steatohepatitis (NASH) by co-culturing HepaRG cells with macrophages and stellate cells. Indeed, fatty liver can progress in a significant proportion of patients to NASH, a diseased state that might further favor drug-induced hepatotoxicity via oxidative stress and mitochondrial dysfunction [42,48]. Secondly, although the molecular network allows for an efficient exploration of the drug metabolism, it cannot be excluded that some minor metabolites were not detected with our methodology. Thirdly, it would be interesting to study an independent cohort of COVID-19 patients treated with HCQ, in particular to confirm our data suggesting that DCQ (M2) might favor hepatic cytolysis. Overall, we showed that the HepaRG cell model coupled to molecular networking has proven useful to investigate HCQ metabolism and toxicity in the context of obesity-associated fatty liver. The development of cellular models relevant to other pathophysiological conditions such as hypoxia or inflammation would be of particular interest to better understand the potential changes of drug metabolism and toxicity occurring in the COVID-19 population.

## 4. Materials and Methods

### 4.1. Material

William’s E medium was purchased from Gibco (ThermoFischer Scientific, San Jose, CA, USA). Penicillin-streptomycin was obtained from Life Technologies (Grand Island, NY, USA). Fetal Bovine Serum (FBS) was purchased from Eurobio (Courtaboeuf, France) and from Hyclone^TM^ GE Healthcare Life Sciences (Logan, UT, USA). Hydrocortisone hemisuccinate was purchased from Serb (Paris, France). Dimethyl sulfoxide (DMSO), formic acid, ammonium acetate, insulin, HCQ, neutral red powder, DAPI, Nile red, and Phosphate Buffered Saline (PBS) were obtained from Sigma-Aldrich (Saint Louis, MO, USA). Methanol, acetonitrile, and water were liquid chromatography-mass spectrometry (LC–MS) grade quality and were purchased from Fisher Scientific UK (Loughborough, Leicestershire, UK). 2,3-Bis(2-methoxy-4-nitro-5-sulfopheny)-2H-tetrazolium-5-carboxyanilide inner salt (XTT) and phenazine methosulfate (PMS) were purchased from SERVA (Heidelberg, Germany) and TCI (Zwijndrecht, Belgium), respectively.

### 4.2. Cell Culture and Treatment

The human renal proximal tubule epithelial cell line RPTEC was purchased from ATCC (LGC Standards, Molsheim, France). The cells were cultured in DMEM/F12 supplemented with hTERT Immortalized RPTEC Growth Kit (LGC Standards, Molsheim, France). Briefly, RPTEC were seeded at a density between 1.2 and 1.8 10^5^ cells/well in 96-well plates in supplemented culture medium. The culture medium was refreshed every day until the cells reached confluence, prior to utilization.

Progenitor HepaRG cells were cultured, as previously described [29]. Briefly, HepaRG cells were seeded at a density of 10^5^ cells/well in 96-well plates in a culture medium William’s E medium (1×) supplemented with 10% FBS, 50 U/mL penicillin, 50 µg/mL streptomycin, 5 µg/mL insulin, 2 mM glutamine, and 50 µM sodium hydrocortisone hemisuccinate. After 2 weeks, the cells were cultured during two additional weeks in the same medium supplemented with 2% DMSO. After 4 weeks, the HepaRG cells were differentiated in both cholangiocyte- and hepatocyte-like cells [49].

For acute cytotoxicity, differentiated HepaRG cells and confluent RPTEC were treated for 48 h with HCQ prior to analysis. For chronic cytotoxicity, HepaRG cells were treated with HCQ alone (control condition), or in combination with a mixture of fatty acids (150 µM stearic acid and 150 µM oleic acid) every 2 days for 10 days, as previously described [32,33]. At day 9, the medium was refreshed and the detection of HCQ and its metabolites was performed at the end of the experiment.

### 4.3. XTT Assay

The cytotoxicity of HCQ in HepaRG and RPTEC was assessed by evaluating the mitochondrial function with XTT. Following treatment, the cells were rinsed with PBS and incubated with 100 µL of a solution containing XTT (0.5 mg/mL) and PMS (0.6 mg/mL). After 20 min of incubation, absorbance was measured at 480 nm using a spectrophotometer plate reader. Viability was determined by comparing the absorbance of the tested conditions to the untreated (control) cells. The results were expressed as the mean percentage of cell viability relative to the control cells.

### 4.4. Neutral Red Uptake (NRU) Assay

Neutral red solution (0.1%) was prepared in PBS and stored at 4 °C. Following the XTT assay, the cells were rinsed in PBS and 100 µL of neutral red solution (80 µg/mL) prepared in PBS was added to each well and was incubated 2 h at 37 °C. The cells were then rinsed in PBS, and 100 µL of solubilization solution (1% acetic acid in 50% ethanol) was added to each well. Absorbance was read at 540 nm. Viability was determined by comparing the absorbance of the tested conditions to the untreated cells. The results were expressed as the mean percentage of cell viability relative to the control cells.

### 4.5. Immunofluorescence

After incubation with HCQ, the cells were fixed with 4% paraformaldehyde in PBS and were permeabilized with 0.2% Triton X-100. The plates were then incubated in blocking solution (PBS with 1% BSA and 0.05% Tween-20) for 30 min before the addition of probes. Nuclear DAPI (1 µg/mL) and Nile red (10 pg/mL) were prepared in PBS and were incubated for 30 min. The plates were then washed in PBS and were used for automated cell identification by high content analysis using two channels for excitation/emission wavelengths, namely 531/593 nm for Nile red and 350/461 nm for DAPI. Nile red depicted a polarity-dependent fluorescence spectrum from 550 nm (non-polar lipid) to 650 nm (phospholipids) [34].

### 4.6. High Content Analysis

The plates were scanned with the Thermo Scientific ArrayScan VTI HCS Reader (Thermo Scientific, Waltham, MA, USA) and were analyzed using the Target Activation module of the BioApplication software (Thermo Scientific, Waltham, MA, USA). For each well, 10 fields (10× magnification, white bar = 100 µm) were scanned and analyzed for immunofluorescence signal quantification. Cell viability was determined by cell counting from DAPI staining and was expressed as a percentage of cells compared to the vehicle control conditions. Lipid staining was quantified in the whole cells and was expressed as a fold increase compared to the untreated cells.

### 4.7. Sample Extraction

In vitro (culture medium) and in vivo (plasma) samples (200 µL) were extracted as already described [37]. Briefly, samples were supplemented with 500 µL of methanol containing internal standards and then extracted with 300 µL of 0.1 M zinc sulfate solution. After evaporation of the supernatant, the residue was dissolved in 200 µL of LC-MS grade water and transferred into chromatographic vials for the LC-HR-MS analysis.

### 4.8. LC-MS Settings

LC-MS analyses were performed using an Orbitrap Q Exactive™ mass spectrometer coupled to an UltiMate 3000 pump (Thermo Scientific, San Jose, CA, USA), and a heated electrospray ionization source (HESI-II) was used for the ionization of the target compounds, as already described [27]. Data acquisition, calibration, and instrument control were performed using Xcalibur ^®^ 2.1 (Thermo Scientific) software. Samples were maintained at 15 °C in the autosampler and quality controls were injected before each analysis. The screening LC-HRMS/MS method used for molecular networking building was as follows: The mobile phases were composed of ammonium formiate at 2 mM and formic acid 0.1% in water (phase A) and ammonium formiate at 2 mM and formic acid 0.1% in methanol and acetonitrile (50/50) (phase B). LC was performed on an Accucore Phenyl Hexyl (100 × 2.1 mm, 2.6 µm) (Thermo Scientific, San Jose, CA, USA) using the following gradient elution, as already described [27]: initial conditions of 99:1 (A:B) maintained for 1 min, increased to 1:99 (A:B) for 9 min, followed by a 1.5 min plateau with 1:99 (A:B), and return to initial conditions at 99:1 (A:B) for equilibration. This corresponded to a total chromatographic run of 15 min. The flow rate was 500 µL/min, the column temperature was maintained at 40 °C, and the injection volume was 20 µL. For mass spectrometry, the instrument was operated in electrospray ionization (ESI) positive mode, and the range for acquisition was 220 to 550 *m/z*. Ion precursor selection was performed in a data dependent mode of operation, with the most intense ion from the previous scan selected for fragmentation. An inclusion list was added to ensure that the predicted metabolites were prioritized for fragmentation. Full scan data (MS1) were acquired for each ionization mode at a resolution of 35,000 FWHM, with an AGC target of 1e6 and a maximum injection time of 120 ms. The source parameters were as follows: source voltage + 3.0, sheath gas flow 60 units, auxiliary gas flow 10 units, capillary temperature 320 °C, and S-Lens RF level 60 units. MS/MS (MS2) data were acquired at a resolution of 17,500 FWHM with an AGC target of 1e5, maximum injection time of 50 ms, a TopN of 8 in positive mode, and an isolation window of 2.0 *m/z*. The normalized collision energy (NCE) was scaled at 17.5, 35, and 52.5, and the dynamic exclusion time was set at 3 s.

### 4.9. Molecular Networking Generation

As already reported [27], the spectral data allowed us to generate molecular networking using the semi-quantitative bioinformatics approach. Data acquisition, processing (i.e., MS data conversion, preprocessing, MS1 annotation, and generation of molecular networks), visualization, and network analysis have been described in detail elsewhere [26]. Briefly, raw data were converted to open MS format (.mzXML) with a ProteoWizard’s MSConvert module. The mzXML files were then preprocessed (deconvolution, de-isotoping, alignment, and gap-filling) with MZmine 2 software [50]. The single .mgf output file was then loaded on the Global Natural Products Social networking (GNPS) web-based platform in order to generate the multimatrix molecular network [25]. For the use of high-resolution data, the basic parameters were modified to *m/z* 0.02 for the mass tolerance of precursor and fragment ions used for MS/MS spectral library searching, and *m/z* 0.02 for the mass tolerance of fragment ions used for molecular networking. The minimum cluster size was set to 1. In addition, links between nodes were created when the cosine score was greater than 0.70, and the minimum number of common fragment ions shared by two MS/MS spectra was 6. Links between two nodes were only kept in the network if each node was in the top 10 most similar nodes. The molecular network was visualized using Cytoscape 3.8.0 software [51]. As Cytoscape software allows for the visualization of all the data from the analysis of the samples, including the peak area of the different compounds found, the peak area used in this study was collected at this step. The nodes were annotated by spectral matching in comparison with the online libraries of GNPS, mzCloud, and by information propagation.

### 4.10. Patients

Plasma sample analyses were performed on a cohort of 17 COVID-19 patients admitted in ICU at Rennes University Hospital. Blood samples were obtained from a study approved by the Rennes Hospital Ethic Committee (CHU Rennes, n°35RC20_9795_HARMONICOV, ClinicalTrials.gov Identifier: NCT04373200), and informed consent was obtained from patients in accordance with the Declaration of Helsinki. All patients received HCQ (Plaquenil^®^) treatment of 400 mg/day until day 4 (D4) post-admission. At D4, the peripheral blood was collected in tubes containing lithium heparin and the plasma samples were prepared and stored at −80 °C prior to the HPLC MS/MS analysis.

### 4.11. Statistics

All of the experiments were repeated at least three times. One-way analysis of variance (ANOVA) followed by Dunnett’s post hoc tests were performed using Prism 8 (GraphPad Software, Inc., La Jolla, CA, USA). All of the error bars denote standard error of the mean (SEM). Statistical significance was depicted as follows: * *p* < 0.05, ** *p* < 0.01, *** *p* < 0.001. Pearson correlation coefficients between patients’ characteristics and relative quantification of HCQ and its metabolites were calculated using Microsoft Excel.

## Figures and Tables

**Table 1:** Patients’ characteristics.

Characteristics	
Patients D0/D7 n	17/17
Age, median IQR	57 (54–67)
Male, *n* (%)	12 (70)
ICU, Clinical Ward, n	17/17
Length of stay in ICU (days), median IQR	14 (6.5–21.5)
Length of stay of hospital (days)	19 (10–23)
**Comorbidities**	
BMI (kg/m^2^), median IQR	29 (27.0–32.5)
Diabetes, *n* (%)	3 (17.6)
Cirrhosis, *n* (%)	0 (0)
Chronic kidney disease, n (%)	2 (11.8)
**Severity criteria and events occurring during follow up**	
PaO2/FiO2 at D4, median (IQR)	200 (174–264)
Renal failure, *n* (%)	8 (47)
Death, *n* (%)	2 (11.8)

D: day; IQR: interquartile range; ICU: intensive care unit; BMI: body mass index; PaO2/FiO2: ratio of partial oxygen pressure to inspired oxygen fraction representing an index of severity of hypoxia (the lower the ratio, the more severe the disorder).

**Table 2:** Pearson correlation coefficients between patients (*n* = 17) biological characteristics and HCQ metabolites.

	Ratio M1	Ratio M2	Ratio M3	Ratio M4	Ratio M5
Age	0.25	−0.18	0.03	0.21	0.06
BMI	0.03	0.03	0.11	0.02	0.02
Temperature	−0.56	−0.59	0.32	0.41	0.15
P/F	0.21	0.08	−0.24	−0.44	−0.44
Cortisol	−0.11	−0.10	0.19	0.30	−0.01
PaO_2_	−0.25	−0.15	0.57	0.38	0.90
Lactate	0.50	0.14	0.02	0.28	0.51
Urea	−0.30	−0.10	0.67	0.80	−0.04
Creatinine	−0.21	−0.06	0.69	0.95	0.07
CRP	−0.27	−0.34	−0.04	0.11	−0.27
PCT	−0.16	−0.24	0.51	0.37	0.71
Bilirubin	−0.32	−0.24	0.07	0.51	−0.23
AST	0.01	0.50	0.03	−0.10	−0.13
ALT	0.11	0.61	0.06	−0.16	−0.04
ALP	−0.10	0.36	0.22	0.05	−0.07
GGT	−0.22	0.18	0.19	−0.16	−0.12

Metabolite ratios were calculated by dividing the peak area of the metabolites (M1 to M5) by the peak area of HCQ for each patient’s plasma, and were correlated to the biological characteristics using Pearson correlation coefficient. Positive and negative correlations are indicated in green and red, respectively. BMI: body mass index; P/F: PaO_2_/FiO_2_ ratio of alveolar pressure of oxygen to fraction of inspired air; PaO_2_: alveolar pressure of oxygen; CRP: C-reactive protein; PCT: procalcitonin; AST: aspartate aminotransferase; ALT: alanine aminotransferase; ALP: alkaline phosphatase; GGT: γ-glutamyltransferase.

## Supplementary Materials

Supplementary data are available online at https://www.mdpi.com/article/10.3390/ijms23010082/s1.
