# Supplementary material for: Molecular Networking for Drug Toxicities Studies: The Case of Hydroxychloroquine in COVID-19 Patients
Source: Int J Mol Sci. 2021 Dec 22;23(1):82. doi: 10.3390/ijms23010082 (PMC8744768; doi:10.3390/ijms23010082)
Supplement: Supplementary file 1 [file ijms-23-00082-s001.zip › ijms-1504856-supplementary.pdf]

Supplementary Table S1. Observed exact mass shifts and corresponding formula changes (resulting from biotransformation) in the hydroxychloroquine cluster.

**Biotransformation**	**Exact mass shift (Da)**	**Formula change**
Deethylation or 2 x demethylation	-28.0313	$-C_2H_4$
Carbonylation	+13.9793	$-H_2 + O$
Deacetylation + Dehydrogenation	- 44.026	- C2H4O
Glucuronidation	+176.0321	$+C_6H_8O_6$

Supplementary Table S2: Putative identified compounds or metabolites contained in the molecular network.

Observed *m/z* [M+H$^+$]	Theorical *m/z* [M+H$^+$]	Molecular formula	Δ exact mass error	ESI-HRMS² data (*m/z*)	t_R (min)	Putative compound identification
336.1834	336.1842	$C_{18}H_{26}ClN_3O$	0.0008	247.10; 191.04; 179.04; 158.15; 102.09; 90.09; 69.07	3.9	Hydroxychloroquine
512.2156	512.2163	$C_{24}H_{34}ClN_3O_7$	0.0007	336.18; 247.10; 191.04; 179.04; 158.15; 102.09; 90.09; 69.07	4.1	Hydroxychloroquine glucuronide
292.1570	292.1580	$C_{16}H_{22}ClN_3$	0.0010	247.10; 191.04; 179.04; 114.13; 69.07; 58.07	3.9	Desethylchloroquine
308.1520	308.1529	$C_{16}H_{22}ClN_3O$	0.0009	247.10; 191.04; 179.04; 130.12; 112.11; 74.06; 69.07; 62.06; 56.05	3.7	Desethylhydroxychloroquine
350.1625	350.1635	$C_{18}H_{24}ClN_3O_2$	0.0010	247.10; 179.04; 172.13; 128.14; 116.07; 72.08; 69.07	4.4	Hydroxychloroquine carbonyl derivative
468.2258	468.2265	$C_{23}H_{34}ClN_3O_5$	0.0007	336.18; 337.18 ; 247.10; 179.04; 158.15; 102.09; 69.07	4.2	Hydroxychloroquine xylose conjugate

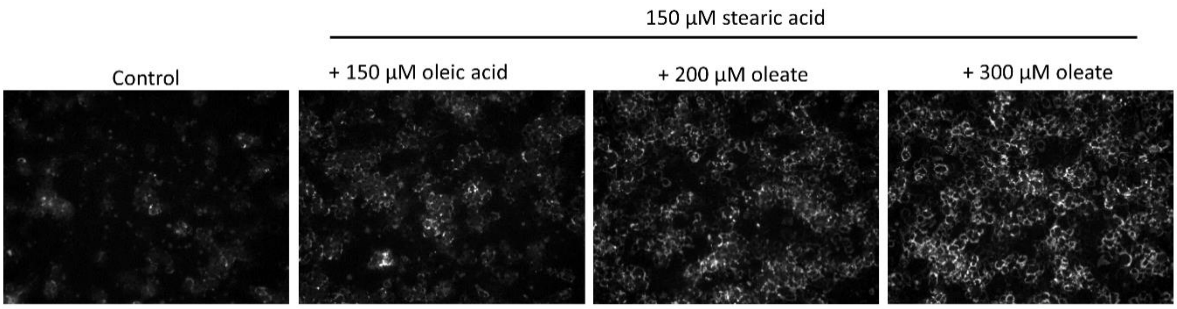

Supplementary Figure S1: lipid staining with Nile Red in HepaRG cells exposed to several mixtures of oleic acid and stearic acid during 10 days (10X magnification).

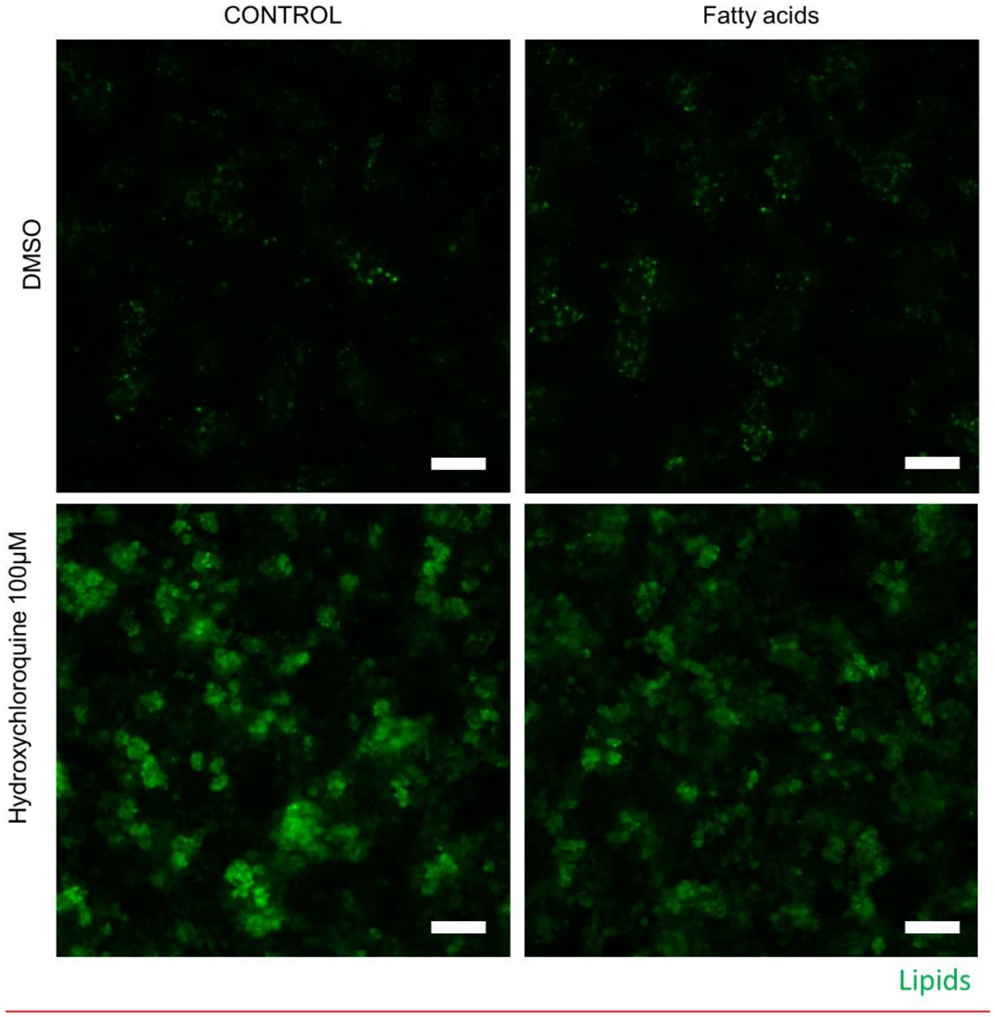

Supplementary Figure S2: Chronic cytotoxicity of HCQ in HepaRG cells exposed to fatty acids. Same representative images at 10× magnification in Figure 3 of HepaRG cells treated 10 days with HCQ and fatty acids. Nile Red in green correspond to cellular lipids. DAPI was not presented in this panel. White scale bar = 100 µm.

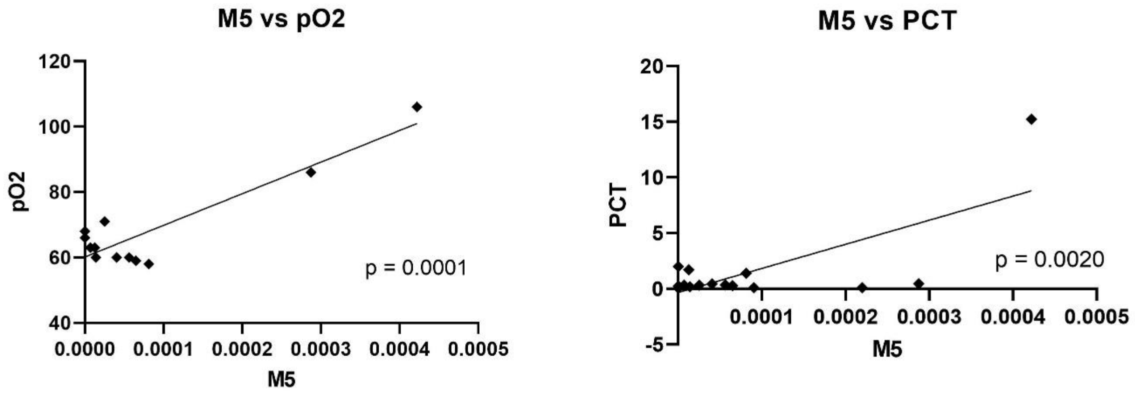

Supplementary Figure S3: Linear regression between metabolite M5 ratio and biological characteristics p02 and PCT. Ratio of the area under the curve (AUC) of metabolite M5 in patients' plasma to the AUC of metabolite M5 in control condition was represented as scatter plot. Patient with missing value for PaO2 at day 4 were excluded of the data set. Linear correlation and p value were calculated using GraphPad Prism.
